# Ex-vivo recruitment and x-ray assessment of donor lungs in a challenging retrieval from a donor supported by lvad using the portable normothermic perfusion system: a case report

**DOI:** 10.1186/s13019-017-0597-1

**Published:** 2017-05-18

**Authors:** Marco Schiavon, Francesca Calabrese, Guido Di Gregorio, Monica Loy, Giuseppe Marulli, Alessandro Rebusso, Fiorella Calabrese, Federico Rea

**Affiliations:** 1 Department of Cardiac, Thoracic and Vascular Sciences, Thoracic Surgery Division, University of Padova, Via Giustiniani 2, 35100 Padova, Italy; 2 Department of Anaesthesiology and Intensive Care, University-Hospital of Padova, via Giustiniani 2, 35100 Padova, Italy; 3 Department of Cardiac, Thoracic and Vascular Sciences, Pathologic Division, University of Padova, via Giustiniani 2, 35100 Padova, Italy

**Keywords:** EVLP, Lung transplantation, Marginal donor, VAD

## Abstract

**Background:**

Lung transplantation (LTx) is limited by the shortage of suitable donors. To overcome this problem, many programs have begun to use donors with extended criteria (marginal donors). However, brain-dead patients with implanted mechanical circulatory support system have rarely been considered as potential lung donors. This case demonstrates the feasibility of lung transplantations from organ donors supported by a mechanical circulatory support system despite the possible difficulties of lung retrieval.

**Case presentation:**

Our case presents a successful procurement and bilateral lung transplantation from a donor supported by a left ventricular assist device (LVAD) who experienced an intraoperatively haemodynamic complication. The use of portable normothermic perfusion device let us to reduce ischemic injury and assess these marginal donor lungs helping us to determine the clinical suitability for transplantation. Given our extensive experience with the device instrumentation and management, the EVLP process was uneventful with excellent post-transplant course.

**Conclusions:**

This case report demonstrates the feasibility of lung transplantations from organ donors supported by a mechanical circulatory support system using the portable normothermic perfusion platform to assess and preserve these donor lungs.

## Background

Lung transplantation represents the gold-standard therapy for patients with end stage lung disease. However, only about 15% of lungs from multiorgan donors are used for transplantation even if the selection criteria for donor lungs have been extended [1, 2]. For many years, brain dead patients with implanted mechanical circulatory support system have not been considered as potential lung donors. Recently, three cases of lung procurement from a donor with a circulatory support devices were reported [3–5].

In these reports, lungs procurement required a careful anatomical preparation of heart and lungs because many surgical difficulties during retrieval might occur.

When this happens, we may face two different situation: on the one hand the risk of not being able to fully assess the suitability of the organ, on the other side a lung deterioration which may impair its function.

Nowadays the use of Ex Vivo Lung Perfusion (EVLP) platform to question and evaluate marginal lungs is part of clinical practice as demonstrated by several studies [6–8] even if to our knowledge no cases of EVLP procedure were reported in this kind of donors.

In our case, we report and confirm the possibility to use lungs from a donor supported by a left ventricular assist device after ex-vivo recruitment and assessment using the Organ Care System (OCS) Lung (Transmedics, Andover, MA, USA) platform in case of difficult retrieval.

## Case presentation

**Fig. 1:**
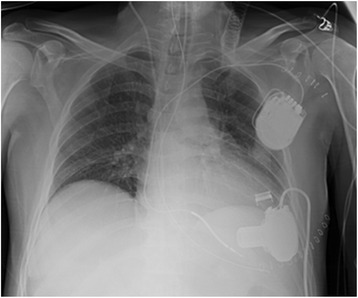
Chest X-ray of the multiorgan donor with an implanted LVAD one day before the donation

A 54-year old male, brain dead donor was evaluated and assigned to our centre for organ donation by the North Italian Transplant program (NITp), our network for organ sharing. The donor was recovering in our hospital post-LVAD implantation (HeartWare®, HeartWare International, Inc., USA) as a bridge to heart transplantation for dilated ischemic cardiomyopathy (Fig. 1).

Cardiac catheterization, performed before the VAD implantation, showed pulmonary hypertension with a mean pulmonary arterial pressure of 42 mmHg, a wedge pressure of 38 mmHg, and normal arteriolar resistance (1.2 HRU).

The cardiac procedure was performed through left anterior thoracotomy with insertion of outflow cannula in the left axillary artery. The post-operative course was uneventful for 16 days when a subarachnoid haemorrhage occurred causing brain death.

The donor was intubated 3 days prior the retrieval and the PaO2/FiO2 ratio (FiO2 100%) at the moment of the call was 511 mmHg. The chest x-ray showed reduced bilateral lung expansion, bronchoscopy was normal, without signs of current infection or aspiration. Eurotransplant donor score [9] was 10.

After a median sternotomy, we proceeded with special care to the inspection of thoracic organs. At the opening of the left pleura, we observed the presence of strong adhesions between the left lung and the outflow cannula. Gentle and careful preparation was required to avoid lung injuries and bleeding complication. At the inspection, the donor lungs did not present signs of infiltrations with a slight degree of edema in the lower lobes, the PaO2/FiO2 ratio was 583 mmHg (FiO2100%, PEEP: 5 cm H20, Tidal Volume: 6 ml/Kg/min) and, therefore, we start to prepare the operative room for transplantation.

Abdominal procedures for liver and kidneys retrieval started but during these manoeuvres, several episodes of severe hypotension occurred, poorly responsive to drugs with risk of sudden right ventricular failure. During a new inspection, lung appeared heavier in comparison to before, mainly due to a small increase of lung edema. In addition, the anesthesiologist was in doubt about the risks of new hypotensive episodes in the donor.

Therefore, considering the risk of organ hypoperfusion and potential lung deterioration from RV failure, we decided to proceed immediately to retrieval with using the OCS lung to perform ex-vivo assessment of these lung and also to minimize donor lung ischemia since the patient was not yet in the operative room.

The OCS was prepped and primed simultaneously with the surgical procurement of the donor lungs in around 20 min. Following injection of heparin, pulmonary cannulation and aortic cross-clamping, the LVAD was deactivated by cutting the driveline. Perfusion with 5 l of Celsior® (4 l anterogradely supplemented with 50 mg nytroglicerine and 1 l retrogradely) and explantation of the double lung block followed standard protocols. After 53 min of instrumentation time, the lungs were connected to OCS Lung starting normothermic perfusion. Concurrently recipient preparation was interrupted waiting for a more extensive evaluation of lungs function through OCS device.

**Fig. 2:**
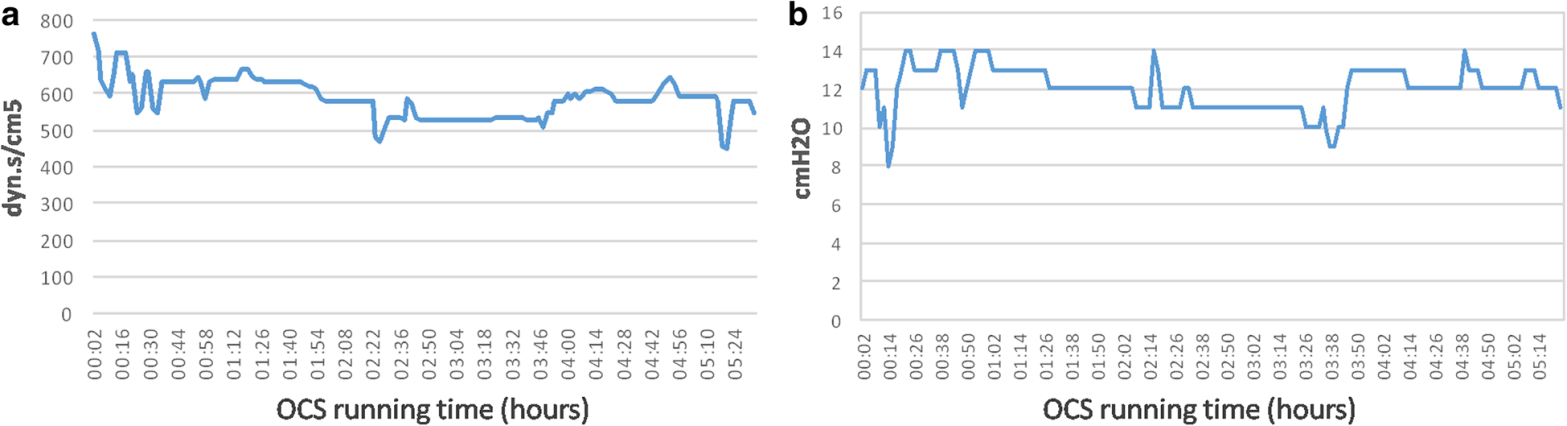
Overtime pulmonary vascular resistance (**a**) and peak airway pressure (**b**) trend during OCS running time showed an improvement/stability of lung parameters during normothermic perfusion

**Fig. 3:**
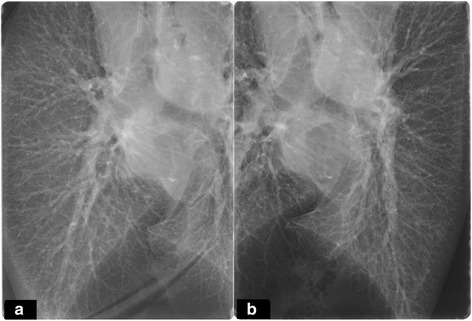
Lung X-ray in the OCS device showing no sign of oedema, infection or other abnormalities in the right (**a**) and in the left lung (**b**)

After 340 min of OCS Lung running time, arterial blood gas (PaO2/FiO2 from 476 mmHg to 525 mmHg) analysis, visual and bronchoscopic evaluation, arterial pulmonary pressure (mean value 11 mmHg), pulmonary and airway resistance (Fig. 2a, b) improved and were satisfactory. In addition, a lung-x-ray on the device was performed, excluding any abnormalities (Fig. 3a, b).

At this point, the anaesthesiological preparation of recipient restarted and lungs were prepared for implantation. The recipient was a 61 year-old male affected by idiopathic pulmonary fibrosis without pulmonary hypertension.

Bilateral lung transplantation was performed via clamshell incision for the presence of pleural adhesions; no extra corporeal membrane oxygenation was needed. Total cold ischemic time was 183 min for the right lung and 292 min for the left side.

In Intensive Care Unit, the severity of primary graft dysfunction (PGD) was calculated according to International Society for Heart and Lung Transplantation (ISHLT) recommendations [10] and resulted respectively 3,2,2,0 at T-0, T24, T48 and T72.

The patient was extubated on day 3 and discharged on postoperative day 33 with an uneventful post-operative course.

Scheduled trans-bronchial biopsy performed before discharge resulted positive for mild acute cellular rejection without bronchiolar involvement (grade A2B0) [11]. The patient was treated with high-dose intravenous steroid and discharged from the hospital with a satisfactory chest X-ray and arterial blood gas exchange.

To date, after 12 months follow-up, the patient is alive with satisfactory condition, without sign of acute rejection at the trans-bronchial biopsies.

## Discussion and Conclusions

The organ shortage remains a key limiting factor to the widespread application of lung transplantation [1]. Among the options available to increase the donor pool, the use of marginal or extended donors [12, 13] is certainly one of the most pursued and adopted. Brain-dead patients with an implanted LVAD have rarely considered as potential lungs donors since the presence of prior cardiopulmonary surgery was usually considered as a relative contraindication to pulmonary donation [14]. The presence of mechanical circulatory support may be associated with different serious adverse events, including infection, bleeding, thromboembolism, neurologic disorders, right ventricular failure and multi-organ dysfunction resulting in poor consequences for pulmonary donation [15].

In addition, patients needing LVAD implantation present a poor cardiac function with presence of pulmonary hypertension, previous episode of lung edema and risk of organ damages.

However, the role of mechanical support for both acute and chronic heart failure is rapidly growing and this donor population will certainly increase in the coming years. To our knowledge, successful transplantation of lungs from a donor with mechanical circulatory support has been limited [3–5]. In these reports, donors were younger than our borderline age donor and the duration of LVAD support before retrieval varies from 1 day to 1 year after implantation.

The retrieval procedure may be, in these cases, most critical. Cardiac redo operations after LVAD implantation are high-risk procedures, due to the presence of the device itself and dense pleural adhesions on the left side that could increase the risk of complications. Differently from the other studies where no anaesthesiologic issues were reported during retrieval, our case was further complicated by severe hemodynamic instability resulting in severe RV failure, that risked compromising our ability to use these lungs as demonstrated by the increase in lung edema at the inspection. Therefore we decided to immediately retrieve the organs using the OCS device with two aims: organ revaluation and reduction of ischemia.

Thanks to the OCS device, we were able to evaluate functional and hemodynamic parameters of the lungs. In particular, despite the donor history of pulmonary hypertension, related to left cardiac failure before LVAD implantation, mean pulmonary artery pressure and vascular resistance were normal during normothermic perfusion, with an excellent transplantation outcome.

In addition to standard clinical EVLP indices, we also performed a radiographic assessment that is particularly useful to carefully exclude the presence of any unrecognized inflammatory/infectious processes that can complicate the post-operative outcome [16].

Compared to static EVLP system, the OCS device has the benefit to monitor lung function since the beginning of normothermic perfusion avoiding any cold transport, that may further compromise lung viability.

The policy of our surgical team is to usually bring the OCS system device in all lung procurement, since some negative findings may be observed during retrieval (technical issues, organ deterioration,.,.).

To assess and to categorize donor lung quality, we generally use Eurotransplant donor score [9]. Despite a donor score above 6 generally indicates a marginal donor, as in our experience, a precise cut-off to discharge donors is not present but it depends on single parameters and on the combination of them. Nevertheless, the score records may prospectively lead us to better understand the impact of donor characteristics on post-operative results.

Moreover, the use of the OCS Lung and its ability to minimize ischemic injury enabled us to time and coordinate the recipient’s procedure. As we previously mentioned the recipient was not in operating room, so the estimated cold ischemia time was higher than 6 h. Prolonged ischemic time has been suggested as a risk factor for the development of PGD [17–20]; the response to ischemia of the organ is highly dependent on many conditions including age and underlying lung conditions at the time of retrieval [21]. While standard donor lungs can tolerate very well up to 8–12 h of cold static inflation with low rates of PGD, the alterations in a marginal organ (i.e. edematous lungs, LVAD implantation, and previous pulmonary hypertension) may result in a higher risk of graft dysfunction. In our case PGD score naturally favorable evolved toward a score of 0 at 72 h.

Finally, the avoiding of cold ischemia should be particularly pursued when donor and recipient are in the same hospital as the time for organ transport is absent.

In summary, this case demonstrates the practicability and technical feasibility of lung donation despite an implanted LVAD system supporting the donor’s failing heart. We report the use of the portable of OCS EVLP system as an aid to preserve and assess these marginal donor lungs especially in urgent retrieval situation. Brain dead patients with an implanted mechanical circulatory device should be seriously considered and evaluated for lung donation to potentially expand donor pool and give a transplant possibility to our recipient.

## Abbreviations

ABG: Arterial blood gas
EVLP: Ex-vivo lung perfusion
FiO2: Fraction of inspired oxygen
ISHLT: International Society for Heart and Lung Transplantation
LTx: Lung transplantation
LVAD: Left ventricular assist device
NITp: North Italian transplant program
OCS: Organ care system
PaO2: Pressure of arterial oxygen
PGD: Primary graft dysfunction
